# Changing Personal Values through Value-Manipulation Tasks: A Systematic Literature Review Based on Schwartz’s Theory of Basic Human Values

**DOI:** 10.3390/ejihpe12070052

**Published:** 2022-06-28

**Authors:** Claudia Russo, Francesca Danioni, Ioana Zagrean, Daniela Barni

**Affiliations:** 1 Department of Human Sciences, LUMSA University of Rome, 00193 Rome, Italy; i.zagrean@lumsa.it; 2 Faculty of Education, University Centre of Family Studies and Research, Catholic University of Milan, 20123 Milan, Italy; francescavittoria.danioni@unicatt.it; 3 Department of Human and Social Sciences, University of Bergamo, 24129 Bergamo, Italy; daniela.barni@unibg.it

**Keywords:** personal values, value change, Schwartz’s theory of values, value manipulation, systematic literature review

## Abstract

According to the Theory of Basic Human Values, values are relatively stable, but not immutable, abstract goals which strongly influence peoples’ lives. Since their relative stability, psychosocial research is attempting to understand the extent to which it is possible to induce a voluntary change in people’s personal values. The main aim of this study was to systematically review the existing literature on experiments to induce a value change, also highlighting the theoretical perspectives used to develop the experimental tasks. We conducted a literature search of five databases (SCOPUS, ProQuest, PsycINFO, PubMed, and Web of Science). After the screening and the eligibility phase, we included a total of 14 articles (25 experiments). Most of these studies involved university students and adopted a pre-and post-test design, using different manipulation tasks. The results highlighted the possibility of inducing a voluntary value change, assessed in terms of mean levels and/or rank order. These findings provide new insights regarding the stability of values in the light of the Theory of Basic Human Values. The practical implications and future research directions are discussed.

## 1. Introduction

Values are a topic of great interest in the social sciences and are considered to be a fundamental construct in almost any field of psychology, including social, developmental, clinical, and organisational psychology [1,2,3,4,5]. In social psychology, personal values have been defined by several authors as people’s beliefs about what is desirable and worthy [6]. They are relatively stable across time, situations, and contexts [7,8,9,10,11] but not immutable. As a matter of fact, recent studies have shown how a person’s values can spontaneously change across the lifespan or different contexts [12] or voluntarily in response to an intervention [13]. Most of these studies refer to the Theory of Basic Human Values [10], which is currently the most well-known evidence-based theory on values [14,15]. This theory identifies 10 basic values, each characterised by a different motivational goal: *power* (social status, dominance over people and resources), achievement (personal success according to social standards), *hedonism* (pleasure or sensuous gratification for oneself), *stimulation* (excitement, challenge, and novelty), self-direction (independence of thought and action), *universalism* (understanding, tolerance, and concern for the welfare of all people and nature), *benevolence* (preserving and enhancing the welfare of people to whom one is close), *tradition* (respect and commitment to cultural or religious customs and ideas), *conformity* (restraint of actions that may harm others or violate social expectations), and *security* (safety and stability of society, relationships, and the self).

More recently, to improve the explanatory power of his theory, Schwartz and colleagues [16] proposed a refined set of 19 basic values, which cover the same motivational continuum of the previously theorised 10 values. All the values are inter-related, and their relations can be organised into a circular structure. Values located close to each other in the structure share motivational goals (e.g., universalism and benevolence share the interest of other people’s well-being); on the contrary, values located far away from each other pursue conflicting motivational goals (e.g., benevolence and power). The relationships among values can also be summarised in terms of a two-dimensional structure [10,16]. The first dimension contrasts *conservation* values, which emphasise self-restraint, the preservation of traditional practices, and safeguarding stability, to *openness to change* values, which emphasise the relevance of change, independence, and freedom. The second dimension captures the conflict between *self-enhancement* values, which emphasise the pursuit of one’s own interests and relative success and dominance over others, to *self-transcendence* values, which emphasise concern for the welfare and interests of others. Moreover, Schwartz [17] categorised these value dimensions based on their focus (personal vs. social) and their orientation (self-protective vs. self-expansive). Accordingly, *self-enhancement* and *openness to change* values share a personal focus, while *self-transcendence* and *conservation* values share a social focus. Finally, *conservation* and *self-enhancement* are labelled self-protective values because they are, respectively, oriented to avoiding conflicts and maintaining the social order or oriented to actively control others and situations. On the contrary, *openness to change* and *self-transcendence* are labelled self-expansive values because they express anxiety-free motivations, promoting a flourished growth.

Schwartz’s value structure [10,16] has been widely tested in cross-cultural research [18,19,20,21]. Findings show that values are universally recognised, have a near-universal meaning and that the above-mentioned structure is similar across culturally diverse groups. Nevertheless, individuals and groups can substantially differ in the importance they attribute to each value [22].

The more important a given value is to a person, the more he/she is likely to act in ways that promote the attainment of that value. Values work as a compass in people’s lives. Many scholars have been looking to understand whether and to what extent personal values are related to behaviour [23,24,25,26,27]. The results have consistently shown the role of values in predicting behaviour, although the value–behaviour link is often mediated by attitudes [28,29,30,31] or moderated by individual [32] or contextual variables [2]. To understand the relationship between values and behaviour, it is necessary to take four main processes into account. Firstly, values may affect behaviour only if they are activated [33]. Value activation usually arises from value-relevant environmental features that make a value salient; this can be a conscious or unconscious process which occurs partially independently of our own value priorities [20]. For example, a song or a movie can activate the value of hedonism even if we do not consider hedonism to be important in our life. Second, value priorities guide our attention, perceptions, and interpretation of life events and issues. Third, values work as motivational forces [34]; as such, the most attractive behaviours are those relevant to the inherent goals that values promote. Finally, values play a central role in planning daily actions (e.g., food choice; [35]) as well as broader behaviours with inherent lifelong implications (e.g., choosing a profession; [15]). Consistency between personal values and actions promotes greater subjective wellbeing and self-esteem, and this may lead to an increased likelihood of behaving in a value-consistent manner [2,36]. The circular structure of values theorised by Schwartz [10,17] implies that when a value motivates coherent behaviour, the value on the opposite side of the circle is inhibited [21].

### Can Personal Values Change or Be Changed?

As mentioned above, values are relatively stable during the lifespan from adulthood [7] and across situations and contexts [10,12,37]. To explain this stability, it is crucial to take into account the inherent desirability of values: they represent universally desirable goals, reflecting what people consider important. Indeed, they fulfil the three universal human requirements (i.e., biological, interpersonal, and social institutional needs). As such, also the less important value in one’s value hierarchy is considered rather desirable. The other side of this inherent desirability is that people are satisfied with their own values, and as such, they are not motivated at all to change them [38]. However, this might not mean that values are unchangeable. By “value change”, scholars mean a change in the importance ascribed to one or more values (change in value mean level and/or rank order) usually assessed through self-report measures [39].

The major natural changes in value perceptions are recorded during early adolescence and adolescence; these are the most critical phases for value development as it is the time of identity formation [40,41,42]. Daniel and Benish-Weisman [40], in their longitudinal study involving Israeli adolescents, showed that they are likely to increase their endorsement of self-focused values (i.e., openness to change and self-enhancement) and decrease their evaluation of other-focused values (i.e., conservation and self-transcendence). Vecchione et al. [42] also found that openness to change and self-enhancement values increase over the two years of early adolescence. There is evidence for smaller changes from young adulthood, with the stability of values ranking from moderate to high. As reported by Schuster et al. [43] in their recent review, spontaneous changes in the importance given to values tend to emerge during normative life transitions, such as attending university [44,45] or becoming a parent [46], and non-normative life transitions, such as migration [47,48] or war [49].

The relative stability of values largely depends on their closeness to the ideal self. As such, people tend not to perceive their own values as easily changeable and are not usually motivated to voluntarily change them [50]. According to some theoretical perspectives, values can be conceptualised as a central schema [51] or abstract belief structure through which people organise old and new information [52]. Similar to a central schema, values are resistant to change because people are likely to remember value-congruent events vividly and lastingly and re-arrange or value-*in*congruent events instead [53].

However, the possibility of manipulating values in order to induce change has attracted increasing research interest in recent years. In this regard, it is important to first distinguish between the manipulation of values aimed at increasing their accessibility and the manipulation of values aimed at changing their importance. Originally, scholars pointed their attention to value accessibility, conceptualised as the possibility of accessing values from memory in order to activate predictable patterns of value-related attitudes and behaviours [19]. The majority of the studies that attempted to experimentally manipulate value accessibility used priming as manipulative technique and aimed to increase the accessibility of the target values in order to produce a value-consistent behavioural response without measuring the importance attributed to the primed value(s). Priming refers to a technique through which exposure to a specific stimulus activates an individual’s schema, this influencing the way people respond to another stimulus [54]. There are explicit or implicit priming techniques: explicit priming consists of drawing attention to items that reflect the target value, while implicit priming draws attention to items that do not directly reflect the target values but that are related to it [55]. An example of explicit priming is provided by Roccas ([56] Study 2): in the experimental condition (self-enhancement vs. self-transcendence), participants were asked to rate their agreement with four self-descriptive statements that reflect self-enhancement or self-transcendence values. An example of implicit priming is provided by Verplanken and Holland ([33]; Study 2) to prime universalism values; the authors asked participants to solve 20 unscrambling sentences; each sentence consisted of four words related to the environment, and the participants had to formulate a syntactically correct sentence.

This type of intervention assumes that values are more likely to influence attitudes and behaviour when they are highly accessible in memory [56,57]. Priming a value through an environmental cue (e.g., scrambled sentences; [58,59]) makes that value more accessible and, consequently, more likely to influence congruent behaviours [56]. Promising effects of the manipulation of accessibility values on the relationship between values and various behavioural outcomes emerged from the studies. For example, Sagiv et al. [60], by making self-transcendence (vs. self-enhancement) values more accessible, increased the likelihood for a sample of college students to cooperate (or compete) in a virtual game. More recently, Conte et al. [61] highlighted that the relationship between benevolence, universalism, and their coherent behaviour becomes strengthened by increasing the accessibility of these values.

Differently from the studies which focused on the manipulation of values aimed at increasing their accessibility, studies that focus on the manipulation of values aimed at changing the mean level of importance or the rank order of values are still scarce. They distinguished between a temporary change and a long-term change of values. Temporary value change occurs when a cue or event challenges a person’s value priorities. Specifically, some cues/events can activate specific values because there is a link in memory between them. Under certain points, it could be viewed as a sort of network connection: even if some values are not usually considered important for the person, the cue/event can activate these values, making them suddenly more salient. This connection would lead the person to respond to the event coherently with the activated values; hence, if we immediately ask this person to report his/her values priorities, he/she probably would rate the activated values more important than he/she would have undertaken if this cue/event did not ever occur. Long-term value change occurs when the automatic initial change primes the necessity to build an alternative schema, strengthening this new schema with other already existing ones, affecting daily perceptions, attitudes, and behaviours. Although a single event or cue might lead to a long-term change, this is more likely to take place if the event occurs both gradually and repeatedly [39]. The psychological mechanisms underlying temporary or long-term value change remain, however, unclear. With reference to this, Bardi and Goodwin [39] claimed a need for studies that systematically discuss how values may voluntarily change and identify the mechanisms behind this change.

Based on all the above considerations, the present study intends to systematically search and discuss empirical studies that have attempted to induce a voluntary change in personal values using experimental manipulation. Our research questions were: (1) Is it possible to induce a voluntary value change? (2) What are the theoretical frameworks adopted and the manipulation tasks used? (3) Which psychological mechanisms are considered responsible for the change?

We carefully described the studies trying to highlight the theoretical rationale behind the value-change and the related manipulation tasks. In doing so, we focused on studies that refer to Schwartz’s theory of values because of five main reasons. First, values are a wide and complex construct. As such, the related theories are quite different from each other; the inclusion of all of them could cause wrong-findings generalisation (reporting bias; [62]). Second, many studies on value change adopted this theory as their theoretical framework. Third, as already mentioned above, to date, Schwartz’s theory is the most cited in the field of social psychology and is considered an evidence-based theory of personal values [18,63]. Fourth, the theory, which proposes a dynamic structure of relations among values, allows researchers to study the process of value change more systematically. Finally, the given values and relational structure have been successfully tested in many countries, indicating that they may be universal. This universal recognition of Schwartz’s value system facilitates the generalisability of results.

## 2. Materials and Methods

A systematic narrative approach to reporting findings was used here because of the high degree of heterogeneity in the studies with respect to their research aims, methods (tasks and measures), and reporting of outcomes. PRISMA statements (https://prisma-statement.org/, accessed on 29 May 2022) for reporting systematic literature reviews were followed [64]. Compliance with these guidelines was ensured by completing the PRISMA checklist.

### Search, Screening and Selection Strategies

**Figure 1:**
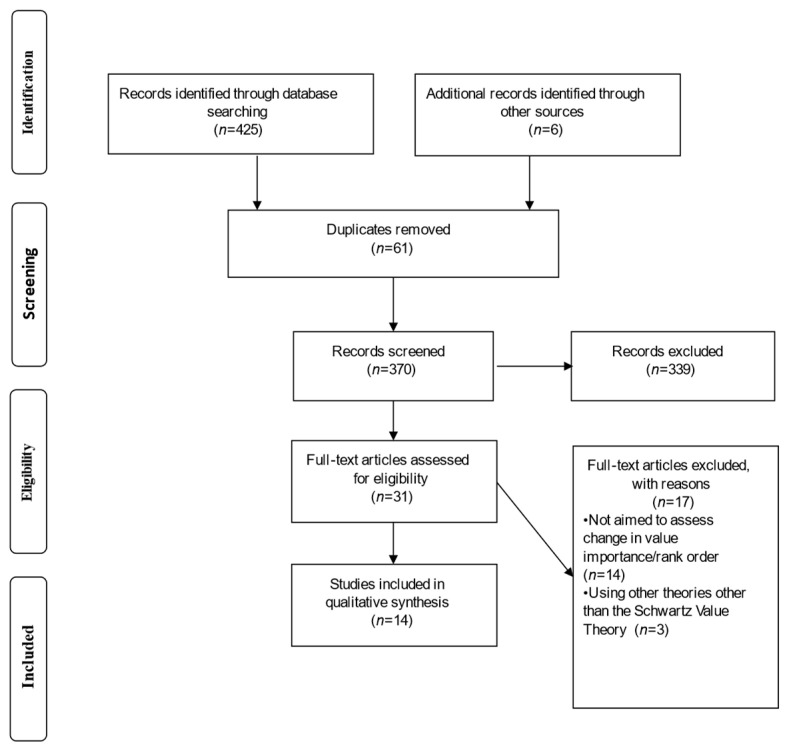
Flowchart for the systematic review procedure.

The first search was conducted in October 2020, with the last update completed on 23 November 2020. Moreover, a second search was conducted in June 2022 in order to eventually include new studies published. We looked for articles describing experiments (or quasi-experiments) that aimed to voluntary change values by using one or more experimental manipulation tasks. The studies were identified by consulting the ProQuest (www.proquest.com, accessed on 29 May 2022), PubMed (https://pubmed.ncbi.nlm.nih.gov/, accessed on 29 May 2022), PsycINFO (https://www.apa.org/pubs/databases/psycinfo, accessed on 29 May 2022), SCOPUS (www.scopus.com, accessed on 29 May 2022), and Web of Science (WOS; www.webofscience.com, accessed on 29 May 2022) electronic databases. Moreover, we screened the reference list of the articles identified through the electronic databases. Figure 1 summarises the steps of the search strategy.

We adopted an iterative search strategy with the combination of the following terms (i.e., Boolean Phrase): (“Personal Values”) AND (“Experiment”) AND (“Value Change” OR “Change of Values”) AND (“Value Importance” OR “Value Enhancement”).

A total of 431 articles were initially identified. During the identification process, we adopted several criteria for the inclusion of in-depth screening: (a) since that 1992 is the year that Schwartz developed his theory, we limited the results to a timespan from 1992 to 2020; (b) peer review research articles in the subject area of psychology written in English or Italian; (c) personal values and value change as the main topics of the articles; (d) with an empirical experimental (or quasi-experimental) design. We excluded: (a) reviews, editorials, letters, case reports, abstracts, conference proceedings, (b) grey literature, (c) studies concerning spontaneous value change (e.g., during life transitions), and (d) research articles from other disciplines unrelated to psychology.

After the removal of duplicates (61 in total), 370 records were screened for their titles and abstracts. After this screening, 339 articles were excluded, and the remaining 31 were further examined by reading the full text. The full articles were obtained for abstracts that met the inclusion criteria. If an abstract was not available or did not present enough information to determine inclusion or exclusion, we obtained the full article and proceeded with full-text screening. During the full screening process, the following eligibility criteria were adopted: empirical experimental or quasi-experimental articles (criterion a) aimed at inducing a value-rating change or a value-ranking change of one or more values through manipulation. By value manipulation, we meant all the tasks employed to challenge value stability and increase the importance of specific values. As such, manipulation tasks can be directly focused on value-content as well as indirectly increasing the importance of the target values by focusing on value-other-related-content (e.g., trying to increase the importance of benevolence by focusing on prosocial content) (criterion b) according to the Schwartz’s Theory (criterion c) and including at least a post-test measure (criterion d).

## 3. Results

### 3.1. Main Characteristics of the Selected Studies

Fourteen articles were included in the present review, for a total of 25 experiments (the screening protocol related to the first systematic search is available as Supplemental Material, https://osf.io/a6qkj/?view_only=b9e4c2f5e95c40209c38cc5040a66984, creation date 21 January 2021).

For each of these experiments, a data extraction sheet was used to summarise the relevant information (authors, year of publication, country, study design and methods, sample description, and type of manipulation). These data were recorded in an Excel spreadsheet and are reported in Table 1.

The fourteen studies included in the review were published between 1998 and 2019. They were conducted in the United Kingdom (4), Israel (3), China (2), USA (2), Australia (1), Germany (1), and Japan (1) and involved adults and young adults or, in one case, adolescents. Eight studies reported a collection of experiments, whereas six studies consisted of a single experiment, amounting to 25 experiments in total. Fourteen experiments sought to manipulate the importance of self-transcendence values, four experiments aimed to manipulate self-transcendence and self-enhancement value importance, one experiment sought to manipulate the importance of self-transcendence and openness to change values, one experiment sought to manipulate the importance of conservation, self-enhancement, and self-transcendence values, and finally, another experiment aimed to manipulate openness to change. Finally, the remaining experiments (N = 4) considered change across the entire value system. Therefore, the majority of the articles included in the review were aimed at increasing the importance assigned to self-transcendence values. These values, which are the goals of child education and positive integration into society, are strongly associated with prosocial behaviours [5,82,83].

As far as the theoretical and conceptual framework was considered, a great heterogeneity among the studies emerged: seven experiments referred more or less explicitly to Bardi and Goodwin’s [39] model of value change; eight experiments tested the value-as-truism-hypothesis [69]; four experiments referred to Terror Management Theory [72]; three experiments referred to the Attachment Theory [75,76]; one experiment tested the dynamic value system hypothesis [80]; one experiment referred to the conceptual beliefs of values and one experiment to the feelings associated to the actualisation of values. All of these frameworks are described below.

Regarding the experimental design, all of the studies adopted a between-subject design, except for the experiment of Howes and Gifford [81], in which a within-subject design was employed. Seven experiments consisted of a post-test design without a pre-test measure of value importance, 16 consisted of a pre- and post-test design, one consisted of a pre-test with a post-test after four weeks, and finally, one consisted of a pre- and post-test with a follow-up test after three months. In all of the studies, except for the within-subject experiment, the participants were randomly assigned to experimental/control conditions, and included a final debriefing session to inform them about the study’s aims and hypotheses. With the exceptions of the experiment by Döring and Hillbrink [67], which was conducted in school classrooms, and the online-based experiment by Howes and Gifford [81], all the other experiments were performed in a laboratory setting where participants took part individually or in small groups. The sample sizes ranged from 4 to 276 participants, with a clear prevalence of females and undergraduate students. The studies used single-item measures related to the target value (e.g., equality), the Brief Inventory of Values (BIV; [84]), the Portrait Values Questionnaire (PVQ; [85]), and the Schwartz Value Survey (SVS; [10]), or an ad-hoc adaptation of these scales.

### 3.2. Theoretical Perspectives and Mechanisms behind the Value Change

We classified the experiments according to the theoretical perspectives adopted to induce the value change, and, starting from the manipulation tasks used by the experiments, we sought to identify the main psychological mechanisms elicited in the value change processes.

#### 3.2.1. The Value Change Model

Bardi and Goodwin [39] developed a theoretical model of value change based on the Elaboration Likelihood Model of Persuasion [86]. Bardi and Goodwin’s [39] model posits the existence of two main routes to value change, one automatic (the peripheral-unconscious route) and one effortful (the central-conscious route). Similar to models of attitude change [86,87], the automatic route of value change involves the activation of primarily automatic processes, while the effortful route requires aware processing for conscious decisions to be made. The process of value change can begin automatically through environmental cues that prime certain values, but to maintain the change over time, the process should be continued through awareness at the cognitive level. Certain environmental cues (e.g., a song) may be associated in memory with one or more values and may prime a certain schema, leading people to use them to interpret future events. However, this priming effect is usually temporary; the automatic route needs repeated stimulation for it to last. Regarding the central route, some environmental cues, for example, a trip, a new job experience, or moving to another city, might lead people to consciously reconsider their value priorities. The outcome should be a stronger relationship between the “revaluated” values and their related coherent behaviour [88,89]. Bardi and Goodwin [39] identified five facilitators of value change: *priming* (the change is caused by environmental features/cues that are associated in memory with certain values), *consistency maintenance* (the change occurs to maintain consistency or preserve a sense of self-coherence), *identification* (the change is due to identification with a significant person and/or a new group), *adaptation* (the change is the consequence of adjusting to a new group and/or culture), and *persuasion* (the change is due to societal influence). The first one, namely priming, mainly works through the automatic-unconscious route to value change. Identification and adaptation mainly work through both the routes, whereas consistency maintenance and persuasion mainly work through the central-conscious route.

##### The Peripheral-Unconscious Route

In this section, we discuss the experiment that relied on stimulating the peripheral route towards value change according to the rationale provided by Bardi and Goodwin’s [39] model. The experiment used implicit priming as a facilitator to induce the value change.

A recent pre/post-test study [65] examined the change in basic values in bi-cultural (Western and Chinese) participants under different culture-priming stimuli (10 culture-priming pictures: Western vs. Chinese). To analyse the effects of the primed stimuli on values, the authors carried out a series of two-way mixed ANOVAs using personal values raw scores. In the case of Western culture-priming, the importance given to personal-focused values (self-enhancement and openness to change) increased (Cohen’s *d* = 0.40), while in the case of Chinese culture-priming, the importance given to social-focused values (self-transcendence and conservation) increased (Cohen’s *d* = 0.43).

##### The Central-Conscious Route

The central-conscious route requires the conscious processing of relevant information to decide on the importance of a target value. As discussed earlier, various mechanisms and facilitators can stimulate this route, all of which support reasoning about values and their importance.

Maio et al. [13] proposed the use of self-confrontation manipulation to change values in the desired direction. Self-confrontation tasks use consistency maintenance as a facilitator [39]. Maio et al.’s [13] experiment consisted of four experimental conditions, one condition for each value dimension—*self-enhancement*, *self-transcendence*, *openness to change*, and *conservation*—to which participants were randomly assigned. After the participants ranked the importance of values, they were shown a fictitious average value ranking of a group of peers where, depending on the experimental condition, each value dimension was ranked as the most important. The participants were required to compare their own value ranking to their fictional peers’ rankings, to read a short essay about the importance of the target value and the characteristics of the people who endorse it, and finally, to write a short personal consideration of why they felt the peers held the target value in esteem. Overall, the mixed-model ANOVAs results revealed that the self-confrontation task led to an increase in the perceived importance of the target values (e.g., self-transcendence) while, at the same time, there was a decrease in the perceived importance of the opposite values (e.g., self-enhancement).

##### The Peripheral-Unconscious and Central-Conscious Routes

Three studies [66,67,68], for a total of five experiments, sought to induce a value change by stimulating both of the routes simultaneously.

Arieli et al. [66] developed an intervention to increase the importance of self-transcendence values. The intervention, which lasted for 30 min, relied on the facilitators of priming, consistency maintenance, persuasion, and self-persuasion and consisted of several tasks: (1) the participants read scientific evidence about the benefits of being other-focused and the percentage of people involved in other-focused activities in order to highlight whether, on average, people are more benevolent than most other people realise (persuasion); (2) the participants completed a prosocial check-list composed of prosocial behaviours that most people frequently adopt (e.g., offering a suggestion to a friend; priming and consistency maintenance); (3) the participants had to recall and write a prosocial-related experience (priming and consistency maintenance); (4) the participants had to write an essay attempting to persuade a panel of reviewers about the importance of being benevolent (self-persuasion). In the control condition, participants completed a similar intervention about personality. The authors tested the effectiveness of the intervention by (1) asking participants to rate on two occasions (pre- and post-intervention) the importance they gave to self-transcendence (Experiment 1), (2) testing the likelihood that the participants would increasingly act in a prosocial way (Experiment 2), and (3) by analysing whether the increase in self-transcendence values remained after four weeks (Experiment 4). The repeated-measures ANOVAs and paired-samples *t*-tests results showed the efficacy of the intervention for increasing both the importance given to self-transcendence values (Cohen’s *d* = 0.53, Power (1 − β) = 0.69) and the likelihood of behaving prosocially (Cohen’s *d* = 0.64, Power (1 − β) = 0.99). The effects of the intervention lasted for more than four weeks (Cohen’s *d* = 0.43, Power (1 − β) = 0.73).

Döring and Hillbrink [67] employed identification as the main facilitator of value change using a set of scenes from the movie *Into the Wild*. The experimental task simply consisted of watching these scenes. The participants in the control condition were asked to complete a puzzle game instead of watching the movie. The participants were asked to complete the PVQ (Schwartz et al., 2001 [85]) before and after watching the movie or completing the puzzle. The *t*-test results highlighted an increase in the importance of stimulation, self-direction, and universalism in the experimental group, although, in contrast, conservation decreased in importance. The changes for universalism (Cohen’s *d* = 0.40) and conservation (Cohen’s *d* = 0.53) were more consistent in the experimental group than in the control group.

Finally, Ma et al. [68], through an experiment consisting of a 180-day space simulation, employed adaptation as the main facilitator of value change. The authors did not formulate specific hypotheses about the direction of the change, but they suggested that the confinement might have caused a decrease/increase in at least some values. After the participants’ recruitment and their training, four subjects enrolled in the “180-Day Controlled Ecological Life Support System Integration Experiment” (CELSS), conducted from June to December 2016. The participants were asked to live in the CELS facility (370 square meters and 1340 cubic meters) for six months: the first (day 1 to day 71) and the third phase (day 109 to day 180) followed Earth’s 24 h cycle, while during the second phase (day 72 to day 108) the 24 h and 40 min cycle was followed in order to simulate the Mars Sol. The participants completed the PVQ-21 a week before their enrolment in the experiment, seven times during their confinement (once for months), and three days after the end of the experiment. The authors conducted nonparametric Spearman’s correlations to investigate the link between times and values, and the paired-sample *t*-tests were to assess differences in mean-levels value priorities during the three phases of the experiment. The results showed that the emphasis on power significantly decreased over the experiment period (r = −0.86, *p* = 0.001), as well as universalism and benevolence mean levels, respectively, decreased and increased during the confinement (universalism Cohen’s *d* = 1.82; benevolence Coehn’s *d* = −2.08).

#### 3.2.2. Values as Truisms

The “values-as-truisms” hypothesis suggests that values are mainly acquired through uncritical socialisation and internalisation processes. Since values are usually widely shared within a specific context, most people do not need to build cognitive support for them because it is unlikely that their values will be challenged within that context [69,90]. In light of this hypothesis, forcing people to analyse the reasons why a value is important or unimportant to them may be an effective way to attack values, namely by causing values to be more vulnerable and changing them [69,70]. Based on previous studies dealing with attitudes [91], Maio [92] suggested that analysing the reasons for holding a given value can cause value change, but only when there is a lack of cognitive support, namely a lack of conscious rationalisation about why some values are more important to the self than others. Thus, for highly-shared social values, such as self-transcendence or conservation values, it is more likely that reasoning causes a change; indeed, the more a value is widespread and socially shared, the less likely it is that people are used to critically reflecting on its importance. Coherently with this approach, value attacks can be hindered by “inoculation”, which is a small attack that forces people to start reflecting on their priorities, making them less truistic. This small attack might allow people to defend their value priorities before a later, stronger attack [90].

In line with the above perspective, in the first of the three experiments carried out by Maio and Olson [69], participants were asked to list their reasons why self-transcendence values are important or unimportant to them. In the second and third experiments, the authors controlled cognitive support for the target values (the presence of arguments supporting the values), the feelings about those values (Experiment 2), and a wide range of individual differences (e.g., dogmatism and self-monitoring; Experiment 3). From the pre- and post-analyses (mixed-model ANOVAs), it emerged that reasoning contributed to increasing the importance given to self-transcendence values. This result was also obtained in the case of low levels of manipulative cognitive support as well as in the case of low levels of self-monitoring.

Following a similar rationale, Bernard et al. [70] analysed the extent to which inducing participants to reason about equality (the target value) helped them to defend the importance of this value against anti-equality persuasive messages. In Experiment 1, the authors asked participants to write down their reasons opposing (refutational condition) or supporting (supportive condition) equality, to read an essay threatening the importance of equality, and finally, to counter-argue the essay. In Experiment 2, they included a preliminary passive defence condition, where participants read 20 reasons for considering the value of equality as important or unimportant; participants then completed the same tasks used in Experiment 1. The results from the one-way ANOVAs showed that, compared to the control group, participants in both conditions (refutational and supportive) attributed more importance to equality (Cohen’s *d* = 0.57; Experiment 1). The results also showed a positive effect of passive defence on the endorsement of equality, as well as a positive effect of active defence (Cohen’s *d* = 0.57; Experiment 2).

Finally, Blankenship et al. [71] speculated that while values are truisms, attitudes are often more resistant to attacks because people tend to build a robust cognitive structure about the reasons behind their importance [93]. Thus, through their experiments, they aimed to indirectly change attitudes by attacking the related values. In Experiment 2 (the Experiment 1 was not related to values), the authors asked half of the participants to read a message attacking the importance of equality, which is part of self-transcendence values in Schwartz’s theory [10], while the other half read a message attacking the importance of affirmative action. After reading the message, the participants reported their favourability towards the value of equality and the extent to which they were confident about their evaluation of equality. Indeed, according to the authors, both the favourability (i.e., how favourably the person has in that values) and the confidence (i.e., the reliability the person feels in that values) are important meta-cognitive indicators of the value importance. In Experiment 3, half of the participants read a message against the utility of the value of equality in society, while the other half of the participants read a message irrelevant to this value. After reading the message, all of the participants reported their favourability and confidence in the value of equality. In Experiment 4, the authors aimed to replicate the previous experiments with another value—the value of freedom (i.e., an openness to change value). The authors asked half of the participants to recall a previous experience in which they felt confident in the value of freedom, while the other half to recall an experience in which they doubted about it. After the manipulation, participants reported their favourability and confidence in the value of freedom. The results of independent *t*-tests showed that, across the second and third experiment, the participants in the value attack condition reported less favourability and confidence; however, in the fourth experiments, the confidence condition (confidence vs. doubt) did not affect the value favourability but only the value confidence.

#### 3.2.3. The Terror Management Theory and the Existential Threats

The Terror Management Theory (TMT; [72]) suggests that existential threats lead people to a feeling of discomfort and anxiety because they become aware of their death—an effect labelled Mortality Salience (MS; [94]). In order to cope with this distress, people are likely to think about aspects of their lives that may endure after their death [95], such as significant personal relationships [96] or a commitment to humanity and nature [97], and can modify their behaviours. In particular, empirical, experimental findings within the TMT framework showed that when participants become aware of their death, they tend to act more prosocially as a result of a defence mechanism against anxiety and fear [95,98].

In line with this rationale and previous research results, Joireman and Duell [73] analysed the effects of making salient and increasing participants’ awareness of their future death (MS) in proself subjects (people who primarily endorse self-enhancement values) and prosocial subjects (people who endorse self-transcendence values), with the aim to identify which of them (proself vs. prosocial) were more likely to rate as more important self-transcendence values after the MS manipulation. In Experiment 1, the participants were divided based on their value priorities (prosocial vs. proself), and then they were randomly assigned to MS (focused on their death) or Dental Pain (DP) conditions (recall toothache episode). In the Experiment 2a, before the MS manipulation, the authors asked a group of participants to read a description of a “bad prosocial person” (i.e., an unpopular student who wanted to help others but was not effective at it) to another group and to read a description of a good proself (i.e., a student admired from peers and focused on his/her goals), while another group did not read any story. The hypothesis was the following: although MS generally increases the importance of self-transcendent values, whether participants were primed with an unattractive prosocial person vs. an attractive proself person, the MS effects would be mitigated, at least for the proself participants. Finally, in Experiment 2b, the authors replicated the previous experiment but with a different story of a good proself and a bad prosocial. Regarding Experiment 1, ANOVA results suggested that the proself participants in the MS conditions (but not in the DP condition) were more likely to endorse self-transcendence values than the prosocial participants (η^2^ = 0.039). This result was also obtained for the Experiments 2a and 2b in the no-story condition (η^2^ = 0.063; η^2^ = 0.066); however, the proself participants in the proself story condition reported lower levels of self-transcendent values after MS manipulation, while MS led to an increase in self-transcendence values for proself participants in the bad prosocial condition (η^2^ = 0.045; η^2^ = 0.034).

Following a similar perspective, Naveh-Kedem and Sverdlik [74] analysed the effectiveness of MS in increasing the importance of benevolence. Their purpose was to test whether people who are more conscious of their life limits are also more prone to hold values that emphasise the importance of other people’s and community’s wellbeing. To increase their death awareness, the participants were asked to think about their future death and describe their feelings aroused by their thoughts and their ideas about what happens after their death. To test their hypothesis, the authors conducted a regression analysis with control variables (i.e., economic status) in the first step and the experimental condition (treatment vs. control) in the second step. The results evidenced a significant effect of MS on benevolence for the experimental group (*f*^2^ = 0.10).

#### 3.2.4. Attachment Theory: The Secure Base Schema

According to the well-known Attachment Theory [75,99], the attachment system, namely a phylogenetic system designed to maintain proximity to significant others, is active throughout an individual’s lifespan. Indeed, the attachment system determines the development of relational schema, namely a cognitive structure in which there is information about the others and the self. This schema is strongly related to people’s proximity-related thoughts and behaviours. Having experienced during childhood a relationship with significant others who were emotional available and supportive facilities the development of a secure base schema [75,76]. As known, a secure attachment style leads to a positive view of the self and others and should have positive implications for the way that people regulate their own welfare as well as the welfare of others [100].

Based on these assumptions, three experiments by Mikulincer et al. [77] focused on the effect of activating a sense of secure attachment to parents on the endorsement of the self-transcendence values (benevolence and universalism). In Mikulincer et al.’s [77] post-test only control group study, participants’ secure attachment style was primed by asking them to watch a pictorial representation of supportive others or recollect personal memories containing a “prototypical sequence of secure-base-script” (which, in this case, consisted of remembering a distressing event during which the child communicates his/her distress and need for support to a significant adult, usually the mother or father, who then gives the support the child needs). To examine the effects of their tasks, for each experiment, the authors conducted a series of individual hierarchical regression analyses (outcome: benevolence and universalism). For all the experiments, the explained variance ranged from 15% to 32% for benevolence and from 25% to 35% for universalism. The results clearly showed that for both the priming stimuli, the contextual activation of a sense of security led participants to perceive self-transcendence values as more important.

#### 3.2.5. Beliefs about Human Values

According to Bain et al. [78], the implicit or explicit beliefs about the nature of values might be a crucial psychological basis of the importance people attribute to them. The rationale of their study underlined Locke’s [101] distinction among beliefs useful to evaluate and categorise objects. Specifically, according to Locke [101], people categorise the objects around them based on the following conceptual belief: natural kinds (e.g., animals and flowers), nominal kinds (e.g., squares), and artefact kinds (e.g., pens and pencils). The first ones are biological entities that are an inherent part of nature; the second ones are conventions and definitions created by a society in order to understand physical objects. Finally, the artefact kinds are human inventions created to pursue an intention. Based on this, Bain et al. [78] suggested that people might have beliefs about human values. As such, they proposed that the value importance might be based on people’s beliefs that values are an essential part of human nature or, on the contrary, they are a social or cultural product useful to organise human existence.

According to the authors’ expectations, the first belief should be strongly positively related to the importance people attribute to values because it entails that the person considers values as inherently part of the human existence, experiencing a sense of volition and freedom in choice. Thus, in their post-test experiment, Bain et al. [78] manipulated the belief about a group of values (i.e., obedience, ambition, power, and helpfulness) in order to lead to higher ratings of these values’ importance. The participants were split into two macro-groups, namely, the belief that a value is a part of human nature vs. the belief that values are a cultural/social product. Within these two macro-groups, the participants were split into obedience, ambition, power, and helpfulness groups.

The authors asked participants to read a description that pointed out the human nature vs. society nature of each value, and then they rated their value priorities. The results of the repeated-measures ANOVAs highlighted that, in the case of human nature condition, values were rated as more important than their opposite values, while, in the case of society nature condition, values were rated as less important than incompatible values. Moreover, a paired *t*-test showed that values were rated as more important when they were described as a part of human nature than a social product.

#### 3.2.6. The Actualisation of Values

The rationale behind Hirose’s [79] experiment arose from Hermans’ [102] and Rogers’ [103] assumptions about the cognitive aspects of values. According to Hermans [102], in the assessment of value priorities, it is essential to take into account the affective components of their endorsement (i.e., the positive or negative feelings associated with the actualisation of values) in order to reveal aspects that would be at risk of otherwise remaining hidden. Similarly, Rogers [103] suggested that people might change their value priorities if they become aware of the cognitive and affective implications of holding a value.

Based on the above considerations, Hirose’s [79] experiment shifted the focus from simple reasoning to the reasoning of feelings and emotions of holding values. Firstly, the participants were asked to rate values as guiding principles in their lives. Secondly, the participants were invited to think about the feelings they could experience if they achieved each value and to rate the degree of pleasure they would experience in actualising each value. Finally, the participants rated the importance of their values, both immediately and after three months from the experimental task. The regression results (pre-test and task-test rates were used as independent and post-test rates as outcome) pointed out an increase in the importance of values immediately post-test. However, the follow-up assessments conducted after three months showed that this increase persisted for only three values (protection of the environment, social justice, and social order).

#### 3.2.7. The Dynamic Value System

From the first value theories, it has always been recognised that the importance given to specific values is relatively stable and largely depends on socialisation processes, cultural influences, and environmental pressures [9,10]. Based on this assumption, Seligman and Katz [80] proposed that the individual value priorities are vulnerable to frequent changes when they are exposed to specific relevant issues.

The authors’ idea was that people have a fluctuating value system: they would not apply their general abstract value priorities within the context of specific issues; rather, when specific issues arise, they modulate and re-evaluate the importance assigned to values. Thus, they proposed a new paradigm, namely the “value dynamics”, alternative to the “value stability” one. Specifically, according to the traditional point of view (i.e., value stability), when an issue is raised for a person, the important values are activated in his/her value system and influence his/her response to the issue. On the contrary, according to the “value dynamics” point of view, people might re-construct their value priorities within a specific context.

In terms of assessment, it means that if we asked people to rank or rate their abstract values, namely the values that work as guiding principles in their lives without any reference to a specific issue, context, or demand, it is likely that these values remain stable over time and situations, but if we ask people to think and rank or rate their values when specific issues arise—with a special concern for the moral ones—it might be possible that there may well be large discrepancies between their abstract values and the concrete ones, namely the values that guide them in the resolution of the issue.

Based on these considerations, in their within-subjects web experiment, Howes and Bain [81] asked participants to read a scenario in which two values were in conflict (protecting the environment vs. pursuing economic gain) and to rate these values in different contexts (i.e., social norms, the immediacy of economic profit, and the immediacy of environmental harm). The mixed-model ANOVAs results highlighted that value importance varied with the scenario, except for the dualist subjects, namely those participants who rated as the two conflicting values as equally important.

## 4. Discussion

A growing body of psychosocial research is interested in understanding whether and how it is possible to voluntarily change personal values. This review systematically analysed studies designed to induce a change in basic personal values conceptualised and measured according to the Theory of Basic Human Values [10,16,20]. Specifically, the main aim of the present review was to highlight the rationale behind the development of value manipulations and discover the mechanisms through which the value change was made possible. Fourteen articles and a total of 25 experiments were included in the review. Most of these experiments were aimed at increasing the importance of self-transcendence values; this is probably because these values are perceived as socially desirable [104], intrinsically part of the prosocial domain [11,32,61], and strictly related to individual and collective wellbeing [105].

Across all the experiments, a significant change in value importance was generally reported, with effect sizes ranging, on average, from small to medium (if given). Thus, referring to the first research question, it is possible to claim that the importance of values can be experimentally manipulated. Regarding the second question, for the development of the manipulation tasks, several theoretical and conceptual perspectives have been employed: the model of value change [39], the value-as-truisms hypothesis [69], the Terror Management Theory [72], the Attachment Theory [75,99], the conceptual belief of values [78], the value actualisation [79], and the dynamic value system [80]. Although these perspectives are very different from each other, they share some common features: firstly, they all have been adopted or developed in order to both manipulate values and explain the induced change in values; these are conceptualised and measured according to the Theory of Basic Human Values [10,17,20]. It is well-known that, within Schwartz’s value conceptualisation, values are guiding principles that are relatively stable across time and situations because they are a fundamental part of personal identity to the point of being considered as a central schema [51]. In line with this, people are usually conservative with regard to their schemas because they are more likely to assimilate new information into already existing schemas rather than developing new ones [52]. Despite this fundamental evidence, the experiments included in the present review provided new insights aimed at challenging this facet of the theory. However, these findings mainly provided evidence for what concerns the temporary change, while the question related to the long-lasting change remains “unsolved” and “unclear”. Secondly, except for the experiments that relied on Bardi and Goodwin’s [39] model, in which the authors explicitly considered the possibility of value change through unconsciousness processes, or Mikulincer’s experiments [77], in which implicit priming was used as a facilitator of value change, all of the others provoked change through a conscious cognitive process. Thus, the change could be viewed as the result of a cognitive re-evaluation.

This evidence is explainable in light of the nature of values: values are mental representations of abstract and desirable goals settled at the highest order cognitive level [19]. Thus, since values are both cognitively represented and, at least in part, cognitively controlled [106], voluntary value change is possible if people find themselves in a position in which they are strained to (re)consider their priorities [15].

Finally, regarding the third research question, we can claim that all the tasks used by the authors raised a significant effect, but as mentioned above, the reported effect size ranges, on average, from small to medium. Only for what concerns the Ma et al. [68] experiment, the effect size was large. Thus, taking into account their results, it can be assumed that the adaptation resulted in the most effective facilitator. Thus, the psychological mechanisms behind the process of adaptation to a new reality seem to be very effective. Nevertheless, in the specific case of the experiment, the authors asked the participants to live in a little cabin for a very long period (180 days in total). This kind of experiment is heavy-handed for participants and, in our opinion, ensues several ethical issues. However, from these findings, the importance of considering a long period of time in order to generate robust value change seems clear. Since values are conceptualised as a central schema, in order to challenge the value stability, it might be crucial to be faced with a new long-lasting situation, as well as, in the case of experiments, to repeat the tasks continually. Nonetheless, the other experiments also reported significant results, and in the case of Arieli et al. [66] and Hirose [79], these results in part lasted for a long period (i.e., four weeks and three months, respectively). These experimental results can become clear in light of the Schwartz Value Theory [10,16,20]. Accordingly, despite their relative stability, values can sometimes spontaneously change: previous evidence, indeed, highlighted that during the main lifespan transitions (e.g., from adolescence to adulthood; [42]) or in case of non-normative events (e.g., in case of war; [49]), people might prioritise the values functional to cope with the new developmental tasks or to adapt to the new situation [43]. Although in the case of the reviewed experiments the change is due to a manipulation, the value change could be interpreted as a functional response to the external demand. Moreover, considering Bain et al.’s [78], Döring and Hillbrink’s [67] and Maio et al.’s [13] experiments, the manipulation tasks elicited an effect also on the opposite values to the target ones, coherently with the circumplex-structure of values theorised by Schwartz [10,16,20]. However, except for two studies [66,79], we cannot establish if changes were short- or long-term. Moreover, in the case of Hirose’s [79] follow-up results, the value change lasted for only three values, namely protection of the environment, social justice, and social order. It is worthwhile noting that this type of long-lasting change, with a specific focus on social justice and social order, might be interpreted in relation to the reform in Japan from 2001 to 2007, when the Japanese Prime Minister approved the expansion of the Japanese Self-Defence Forces. It is possible that during this time, Japanese institutions and policies often used persuasive language to emphasise the importance of justice and social order, thus reinforcing the new schema developed during Hirose’s [79] manipulation.

Other limitations of the studies included in the review must be acknowledged: participants were mostly undergraduate students; this sampling bias reduces the external validity of the experiments and the generalisability of the findings. Furthermore, most of the studies were carried out in laboratory settings. This has a series of advantages, such as a higher internal validity (although it cannot eliminate the influence of external confounding variables; see, [79]); however, since the results indicate a potential for inducing value change, manipulating tasks in daily contexts (schools, families, etc.) would be more relevant. Additionally, only Maio and Olson [69] considered individual differences (e.g., dogmatism) in the process of value change. Not all people are equally open to change [107]. For instance, those who give great importance to openness to change might be more prone to change their schemas [108] than conservative people. Moreover, the need for cognition (the inclination to be engaged in effortful activities) and the need for closure (the inclination to avoid ambivalent information) should be considered when exploring the process of value change according to models of attitude change [86]. Another important point that deserves attention is the ethical implication of value manipulation. For example, the self-confrontation procedure used by Maio et al. [13], or even more the existential threat used by Naveh-Kedem and Sverdlik [74], provoke change in values by inducing a sense of dissatisfaction or negative arousal. Furthermore, in the case of Ma et al.’s [68] experiment, the change is due to a long-lasting confinement period. Thus, it would be important to develop new, less heavy-handed strategies for participants.

Despite these critical points, the selected studies are largely methodologically sound, and the significant results encourage the perspective that personal values can intentionally change. Based on the findings of this review, we believe that it would be important to develop new interventions applied gradually and repeatedly to the daily contexts. Indeed, as a central schema [51], values need to be challenged steadily in order to reach an enduring change. Recently, Russo et al. [32] developed a new brief web-based intervention that was effective in increasing self-transcendence accessibility in a group of adolescents, strengthening the link between these values and consistent behaviours. Therefore, it would be interesting to analyse whether the same intervention repeated over time would also produce a long-term change in the importance of these values. Moreover, we consider extremely promising and innovative to develop tasks that can arouse in memory the association between target values and positive emotions and feelings (e.g., joy or gratitude) in order to induce value change and maybe improve wellbeing. Indeed, according to Schwartz [22], an important but under-investigated feature of values is their link with emotions. For example, a recent study highlighted that by emotion manipulation (in this case, the emotion of awe), self-transcendence values become more accessible, leading to a strengthened relationship between them and prosocial behaviours [109]. Eliciting emotions might, therefore, lead also to an increment in the importance attributed to some values [39]; for example, according to Marini et al. [108], non-anxiety emotions (e.g., gratitude and affection) can foster the importance of anxiety-free values (e.g., self-transcendence). Additionally, in the case of repeatedly-daily-context interventions, the value change could be viewed as a process of learning, and, as known, positive emotions have a strong positive impact on obtaining permanent learning [110].

The findings from this review must be interpreted considering its limitations. Firstly, the study search strategy was restricted to articles published in English or Italian based on the authors’ linguistic competencies. Hence, studies published in other languages were excluded. Moreover, it might be possible that the conclusions reached are affected by publication bias; often, unsuccessful experiments are not published because of the lack of significant results. For this reason, scholars have recently pointed out the usefulness of including grey literature in reviews [111], such as conference papers or non-peer-reviewed sources. However, the inclusion of grey literature should be considered attentively; these types of studies often report preliminary data or can be methodologically incorrect because of the lack of a peer-review process [112]. Moreover, it is noteworthy to point out that a very small number of recent studies have been included in the present review. We believe that this evidence might be due to the fact that, for a long time, personal values have been considered similar to immutable abstract goals [15], leading a very few researchers to challenge their stability, focusing instead on less “obscure” fields of research, such as, for example, the possibility of increasing the value accessibility in order to strengthen their link with coherent behaviours.

## 5. Conclusions

Our findings indicate the possibility of inducing a voluntary value change in people’s personal values and, as such, point to the importance of further studies in this direction in order to enhance interventions that can be used in real-life settings and that are effective in inducing a long-lasting value change. Indeed, we consider it extremely relevant to keep on investigating the possibility of inducing a voluntary value change because it could also foster a consistent positive change in attitudes and behaviour. For example, it might be relevant to develop a “self-transcendence intervention” in schools aimed at preventing transgressive and antisocial behaviour while promoting prosocial ones. In this regard, considering the global COVID-19 coronavirus pandemic, it is evident how important it would have been to develop and implement a self-transcendence intervention’ that could have raise awareness of adolescents on the prevention of COVID-19 to increase their awareness not only of the importance of protecting their health but also that of others around them (e.g., family and friends). Of course, the challenge of building a value change intervention also concerns the field of clinical psychology. For instance, such an intervention could promote the importance of the value of health (including the security of the self) and support psychotherapeutic interventions for mental health issues. In such cases, the application of a value change intervention might increase the patient’s adhesion to therapy and prevent treatment dropout.

## Figures and Tables

**Table 1:** Characteristics of the studies included in the systematic review.

Theoretical Framework	Author(s)	Year	Citation	Country	Study Design	Sample Size	Participants Characteristics	Procedure	Measures Related to Values	Mechanisms (EG)	Tasks (CG)
***Model of value change:*** ***Peripherical route*** [39]	Ye, S., Ng, T.K.	2019	[65]	China	Experiment; pre and posttest design	179	Undergraduate students (134 female, M_age_ = 21.08, SD = 1.54)	Randomisation of participants: Chinese (Biculturals = 28; Sino-centrics = 27; Western-centrics = 28) vs. Western priming group (Biculturals = 36; Sino-centrics = 29; Western-centrics = 31)	PVQ-21	Cultural priming task	No CG
***Model of value change: Central route*** [39]	Maio, G.R., Pakizeh, A., Cheung, W.Y., Rees, K.J. (Experiment 1)	2009	[13]	UK	Experiment: pre-posttest design	175	Undergraduate students (139 female)	Randomisation of participants: self-transcendence group (EG1), Self-enhancement group (EG2), Conservation group (EG3), Openness to change group (EG4); Control Group (CG)	Adaptation of SVS	Self-confrontation—consistency maintenance	Memory task
***Model of value change: Peripherical and central routes*** [39]	Arieli, S., Grant, A.M., Sagiv, L. (Experiment 1)	2014	[66]	Israel	Experiment; pre-posttest design	36	Undergraduate students (20 female, aged 18 to 20)	Randomisation of participants: N_EG_ = 18; N_CG_ = 18	PVQ (pre-test) SVS (post-test)	Priming, consistency maintenance, persuasion, and self-persuasion	Same structure, but with different contents (i.e., personality characteristics)
	Arieli, S., Grant, A.M., Sagiv, L. (Experiment 2)	2014	[66]	Israel	Experiment; pre-posttest design (2 weeks of interval between the two times)	46	Undergraduate students (17 female, aged 19 to 26)	Randomisation of participants: N_EG_ = 24; N_CG_ = 24	SVS (pre-test) PVQ and spontaneous behaviour measures (post-test)	Priming, consistency maintenance, persuasion, and self-persuasion	Same structure, but with different contents (i.e., personality characteristics)
	Arieli, S., Grant, A.M., Sagiv, L. (Experiment 3)	2014	[66]	Israel	Experiment; pre-posttest design after 4 weeks	58	Undergraduate students (28 female)	Randomisation of participants: N_EG_ = 29; N_CG_ = 29	SVS & PVQ (pre-test) PVQ & SVS (post-test)	Priming, consistency maintenance, persuasion, and self-persuasion	Same structure, but with different contents (i.e., personality characteristics)
	Döring, A., Hillbring, A.	2015	[67]	Germany	Experiment; pre-posttest design (one week of interval between the pre and post measure)	154	Female adolescents (84, aged 13 to 15)	Randomisation of participants: N_EG_ = 82; N_CG_ = 72	PVQ	Identification	Puzzle game
	Ma, Q., Sandal, G.M., Wu, R., Xiong, J., Xu, Zi, He, L., Liu, Y.	2019	[68]	China	Experiment; pre and posttest design	4	Adults selected by psychological test (1 female, Mage = 35.5, DS = 6.61)	No randomisation of participants	PVQ-21	Adaptation	No CG
***Values as Truisms*** [69]	Bernard, M.M., Maio, G.R., Olson, J.M. (Experiment 1)	2003	[70]	UK	Experiment; pre-posttest design	75	Undergraduate students (53 female)	Randomisation of participants; ns	Single measure item “equality”	Reasoning	No tasks
	Bernard, M. M., Maio, G.R., Olson, J.M. (Experiment 2)	2003	[70]	UK	Experiment; pre-posttest design (1 to 5 days of interval between them)	70	Undergraduate students (52 female)	Randomisation of participants; ns	Single measure item “equality”	Reasoning	Same structure, but different contents (reasons why liking or disliking some beverages)
	Blankenship, K., Wegener, D.T. (Experiment 2)	2012	[71]	USA	Experiment; pre-posttest design (but only a posttest measure for values)	148	Introductory psychology students (90 female, Mage = 19.7)	Randomisation of participants; two conditions: value attack vs. policy attack	Single item measure of equality	Reasoning	No CG
	Blankenship, K., Wegener, D.T. (Experiment 3)	2012	[71]	USA	Experiment; pre-posttest design (but only a posttest measure for values)	82	Introductory psychology students (32 female, Mage = 19.6)	Randomisation of participants; value attack vs. control	Single item measure of equality	Reasoning	Reading a message relevant to the value of equality
	Blankenship, K., Wegener, D.T. (Experiment 4)	2012	[71]	USA	Experiment pre-posttest design (but only a posttest measure for values)	57	Introductory psychology students (34 female, Mage = 19.7)	Randomisation of participants: doubt vs. confidence	Single item measure of freedom		No CG
	Maio, G.R., Olson, J.M. (Experiment 1)	1998	[69]	UK	Experiment; pre-posttest design	77	Undergraduate students (54 female)	Randomisation of participants; ns	Self-transcendence items from SVS	Reasoning	Same structure, but different contents (reasons for liking or disliking different beverages)
	Maio, G.R., Olson, J.M. (Experiment 2)	1998	[69]	United Kingdom	Experiment; pre-posttest design	138	Undergraduate students (104 female)	Randomisation of participants; ns	Self-transcendence items from SVS	Reasoning	Same structure, but different contents (the same of experiment 1)
	Maio, G.R., Olson, J.M. (Experiment 3)	1998	[69]	United Kingdom	Experiment; pre-posttest design	144	Undergraduate students (105 female)	Randomisation of participants; ns	Self-transcendence items from SVS	Reasoning	Same structure, but different contents (reasons for liking or disliking different beverages)
***Terror Management Theory*** [72]	Joireman, J., Duell, B. (Experiment 1)	2005	[73]	USA	Experiment; pre-posttest design	180	Introductory psychology students (91 female, median age = 19)	Randomisation of participants (two conditions: Mortality Salience [MS] vs. Dental Pain [DP])	SVS (pre) and BIV (post)	Coping strategy to solve Mortality Salience’ anxiety	Dental Pain control group —tasks related to emotions that dental pain aroused.
	Joireman J., Duell, B. (Experiment 2a)	2005	[73]	USA	Experiment; pre-posttest design	231	Introductory psychology students (173 female, median age = 18)	Randomisation of participants (two conditions: MS vs. DP; three story conditions: no story vs. bad prosocial vs. good proself)	SVS (pre) and BIV (post)	Coping strategy to solve Mortality Salience’ anxiety	Dental Pain CG—the same story condition used for EG, but concerned the dental pain
	Joireman, J., Duell, B. (Experiment 2b)	2005	[73]	USA	Experiment; pre-posttest design	265	Introductory psychology students (171 female, median age = 19)	Randomisation of participants: (two conditions: MS vs. DP; three story conditions: no story vs. bad prosocial vs. good proself)	SVS (pre) and BIV (post)	Coping strategy to solve Mortality Salience’ anxiety	Dental Pain CG—the same story condition used for EG, but concerned the dental pain
	Naveh-Kedem, Y., Sverdlik, N. (Experiment 2)	2019	[74]	Israel	Experiment; pre-posttest design	54	Undergraduate students (46 female, Mage = 25.46, SD = 7.18)	Randomisation of participants; ns	PVQ	Coping strategy to solve Mortality Salience’ anxiety	No tasks
***Attachment theory: secure base schema*** [75,76]	Mikulincer, M., Gillath, O., Sapir-Lavid, Y., Yaakobi, E., Arias, K., Tal-Aloni, L., Bor, G. (Experiment 1)	2003	[77]	Israel	Experiment; posttest design	72	Undergraduate students (51 female; aged 20 to 38)	Randomisation of participants: secure base priming condition (N_EG_ = 24) vs. Positive affect priming condition (N_CG1_ = 24) vs. Neutral priming condition (N_CG2_ = 24)	Adaptation of SVS	Secure base priming	Positive affect priming (recall and describe a situation that made them laugh) neutral priming (describe a household situation)
	Mikulincer, M., Gillath, O., Sapir-Lavid, Y., Yaakobi, E., Arias, K., Tal-Aloni, L., Bor, G. (Experiment 2)	2003	[77]	Israel	Experiment; posttest design	60	Undergraduate students (41 female; aged 20 to 35)	Randomisation of participants: secure base priming condition (N_EG_ = 20) vs. Positive affect priming condition (N_CG1_ = 20) vs. Neutral priming condition (N_CG2_ = 20)	Adaptation of SVS	Secure base priming	Positive affect priming (watch funny pictures) neutral priming (watch neutral pictures)
	Mikulincer, M., Gillath, O., Sapir-Lavid, Y., Yaakobi, E., Arias, K., Tal-Aloni, L., Bor, G. (Experiment 3)	2003	[77]	Israel	Experiment; posttest design	66	Undergraduate students (41 female; aged 19 to 40)	Randomisation of participants: secure base priming condition (N_EG_ = 22) vs. Positive affect priming condition (N_CG1_ = 22) vs. Neutral priming condition (N_CG1_ = 22)	Adaptation of SVS	Secure base priming	Positive affect priming (recall and describe a situation that made them laugh), neutral priming (describe a household situation)
***Human Nature Belief***	Bain, P.G., Kashima, Y., Haslam, N.	2006	[78]	Australia	Experiment; posttest design	143	Introductory psychology students (76 female)	Randomisation of participants; two conditions: human vs. society	SVS	Human nature belief	No CG
***Actualisation of Values***	Hirose, H.	2004	[79]	Japan	Experiment; pre-posttest and follow up after 3 months	140	Undergraduate female students (aged 18 to 20)	Randomisation of participants: N_EG_ = 70; N_CG_ = 70	Adaptation of SVS	Reasoning and feelings	Same structure, but participants were asked to rate the degree of inequality of women experience in the actualisation of values
***Value dynamic system*** [80]	Howes, Y., Gifford, R.	2009	[81]	United Kingdom	Online Experiment; pre-posttest design	276	Randomly selected adults and young adults living in western mid-sized Canadian city (66.1% female, Mage = 49.8)	All the participants completed the same task.	SVS (pre) and a part of it (post)	Situational conflicting values issue	No CG

***Note***. EG: Experimental Group; CG: Control Group; N_EG_: sample size of experimental group; N_CG_: sample size of the control group.

## Data Availability

Not applicable.

## Supplementary Materials

The following supporting information can be downloaded at: https://osf.io/a6qkj/?view_only=b9e4c2f5e95c40209c38cc5040a66984.
